# Identification of feature genes and pathways for Alzheimer's disease *via* WGCNA and LASSO regression

**DOI:** 10.3389/fncom.2022.1001546

**Published:** 2022-09-21

**Authors:** Hongyu Sun, Jin Yang, Xiaohui Li, Yi Lyu, Zhaomeng Xu, Hui He, Xiaomin Tong, Tingyu Ji, Shihan Ding, Chaoli Zhou, Pengyong Han, Jinping Zheng

**Affiliations:** ^1^Department of Health Toxicology, School of Public Health in Shanxi Medical University, Taiyuan, China; ^2^The Central Lab, Changzhi Medical College, Changzhi, China; ^3^Collaborative Innovation Center for Aging Mechanism Research and Transformation, Center for Healthy Aging, Changzhi Medical College, Changzhi, China

**Keywords:** Alzheimer's disease, predict model, WGCNA, GEO, bioinformatics

## Abstract

While Alzheimer's disease (AD) can cause a severe economic burden, the specific pathogenesis involved is yet to be elucidated. To identify feature genes associated with AD, we downloaded data from three GEO databases: GSE122063, GSE15222, and GSE138260. In the filtering, we used AD for search keywords, Homo sapiens for species selection, and established a sample size of > 20 for each data set, and each data set contains Including the normal group and AD group. The datasets GSE15222 and GSE138260 were combined as a training group to build a model, and GSE122063 was used as a test group to verify the model's accuracy. The genes with differential expression found in the combined datasets were used for analysis through Gene Ontology (GO) and The Kyoto Encyclopedia of Genes and Genome Pathways (KEGG). Then, AD-related module genes were identified using the combined dataset through a weighted gene co-expression network analysis (WGCNA). Both the differential and AD-related module genes were intersected to obtain AD key genes. These genes were first filtered through LASSO regression and then AD-related feature genes were obtained for subsequent immune-related analysis. A comprehensive analysis of three AD-related datasets in the GEO database revealed 111 common differential AD genes. In the GO analysis, the more prominent terms were cognition and learning or memory. The KEGG analysis showed that these differential genes were enriched not only in In the KEGG analysis, but also in three other pathways: neuroactive ligand-receptor interaction, cAMP signaling pathway, and Calcium signaling pathway. Three AD-related feature genes (SST, MLIP, HSPB3) were finally identified. The area under the ROC curve of these AD-related feature genes was greater than 0.7 in both the training and the test groups. Finally, an immune-related analysis of these genes was performed. The finding of AD-related feature genes (SST, MLIP, HSPB3) could help predict the onset and progression of the disease. Overall, our study may provide significant guidance for further exploration of potential biomarkers for the diagnosis and prediction of AD.

## 1. Introduction

Alzheimer's disease (AD) refers to the onset and development of age-related cognitive and functional decline and specific neuropathology, which Alois Alzheimer first described in 1906 (Pleen and Townley, 2022). Despite numerous efforts over the year, the exact mechanism of AD has not been fully elucidated (Zhang et al., 2021). Studies have shown that immune changes occur in all neurodegenerative diseases, but with significant differences. Evidence indicates that immune cells invade the aging brain and secrete particular substances that disrupt the production of new nerve cells, which could explain the gradual decline in neuronal recruitment during advanced age. This may pave the way for the development of immune strategies against age-related cognitive impairment (Dulken et al., 2019). The study's author, Anne Brunet, also suggested that, even in humans, immune cells enter the aging brain and precisely reach the areas where new neurons are generated. However, much is yet known in this area.

The logic of the present analysis is to integrate the same or similar gene expression patterns into multiple samples and cluster these genes into a module. Then, each module can be related to specific traits or phenotypes and screened to assess sample expression. Key gene regulatory networks can also be explored (Langfelder and Horvath, 2008). In this network, if a gene is well-connected with others, it is called a “highly-connected gene” (hub gene), and subsequent analysis is performed. Moreover, we will define feature genes that could have key roles in the disease and predict its development and progression.

Overall, this study aimed to complement existing AD research by identifying AD-related feature genes through the WGCNA method and finding associations between their expression and immune infiltration.

## 2. Materials and methods

### 2.1. Data source and search

**Figure 1:**
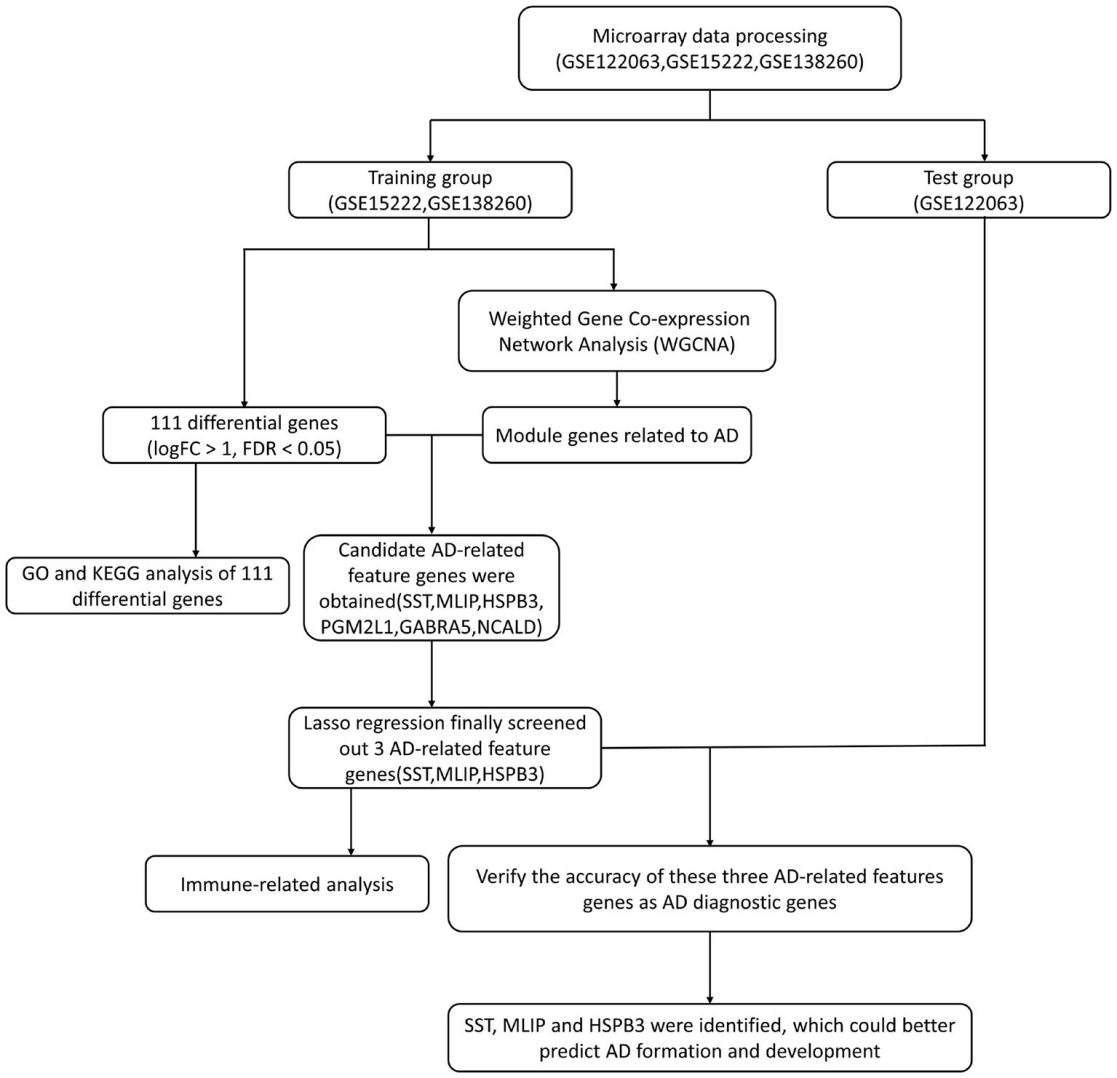
The workflow of data preparation, processing, analysis, and validation.

We retrieved three datasets related to AD from the GEO database (GSE122063, GSE15222 and GSE138260). In these three datasets, there were 249 AD samples and 250 normal samples. Among the AD samples, 56, 176, and 17 corresponded to datasets GSE122063, GSE15222, and GSE138260, respectively. All 499 samples were included in this study. Normal samples were used as control throughout all the study to be able to perform pooled analyses and validate the final model. Moreover, we conducted model verification after its construction. In our method, we merged the datasets GSE15222 and GSE138260 and used the merged data as a training group to build a model. Then, we used dataset GSE122063 as a test group for model validation. A flow diagram of the study is shown in Figure 1.

### 2.2. Differential gene expression

The fusion of datasets GSE15222 and GSE138260 was constructed with the R package termed “sva”. The conditions for screening differential gene expression were set as logFC > 1, FDR < 0.05. According to these stringent criteria, 111 genes were screened and selected for subsequent analysis.

### 2.3. Functional analysis

The Gene Ontology (GO) analysis can identify genes closely related to the origin, development, and phenotypic expression of the disease. In our GO enrichment analysis, three components were included: Biological Process (BP), Molecular Function (MF), and Cellular Component (CC). On the other hand, the Kyoto Encyclopedia of Genes and Genome Pathways (KEGG) enrichment assessment enables systematic gene function analysis by linking genomic and functional information. These two methods were employed to analyze the 111 differential genes obtained before. The R packages involved in the GO and KEGG analysis were “DOSE”, “org.Hs.eg.db”, “clusterProfiler” (Yu et al., 2012), “pathview” (Luo and Brouwer, 2013), and “ggplot2”, which was used to visualize the results.

### 2.4. Network construction and module identification for weighted gene co-expression network analysis (WGCNA)

This analysis was performed using the R package “weighted gene co-expression network analysis” (WGCNA). Briefly, the first step was to cluster samples and then check whether they contain outliers due to human or other reasons. Then, a corresponding weighted correlation coefficient was obtained by measuring each gene, and an adjacency matrix was constructed accordingly. Here, gene similarity should be reflected at both the expression levels and the network topology. However, noise and false positives can affect the adjacency matrix alone. To minimize these issues, in a second step, the adjacency matrix was transformed into a topological overlap matrix (TOM). After that, the genes that had similar expression levels were clustered in modules to be used in subsequent analysis. In a last step, we aimed to identify gene modules associated with AD using Pearson's correlation analysis. This association was adjusted by setting the values of gene significance (GS) and module membership (MM) to 0.4 and 0.8, respectively. The gene modules thus calculated were used as hub genes. Finally, three modules were found to be highly correlated with AD: yellow, green, and magenta.

### 2.5. Screening of AD-related feature genes

After taking the merged dataset as a training group, the screening conditions were logFC > 1, FDR < 0.05, and 111 differential genes were extracted. Then, the genes from the three AD-related modules found in the WGCNA analysis were assessed for intersection. Six matches were obtained (SST, MLIP, HSPB3, PGM2L1, GABRA5, and NCALD) and subsequently filtered by LASSO regression to obtain 3 AD characteristic genes: SST, MLIP, and HSPB3. Lastly, the three were again submitted to validation using the Test group, and all showed high reliability as AD-related feature genes. In addition, we also performed transcription factor analysis of AD-related feature genes, which was done through the HumanTFDB (http://bioinfo.life.hust.edu.cn/HumanTFDB#!/) database.

### 2.6. Evaluation of immune cell infiltration in AD patients

Single sample gene set enrichment analysis (ssGSEA) was performed to quantify the expression of AD-related immune cell subgroups and immune function in normal and AD groups. Then, a correlation analysis was performed between the screened AD-related feature genes and the immune cells.

## 3. Results

### 3.1. Data collection

**Figure 2:**
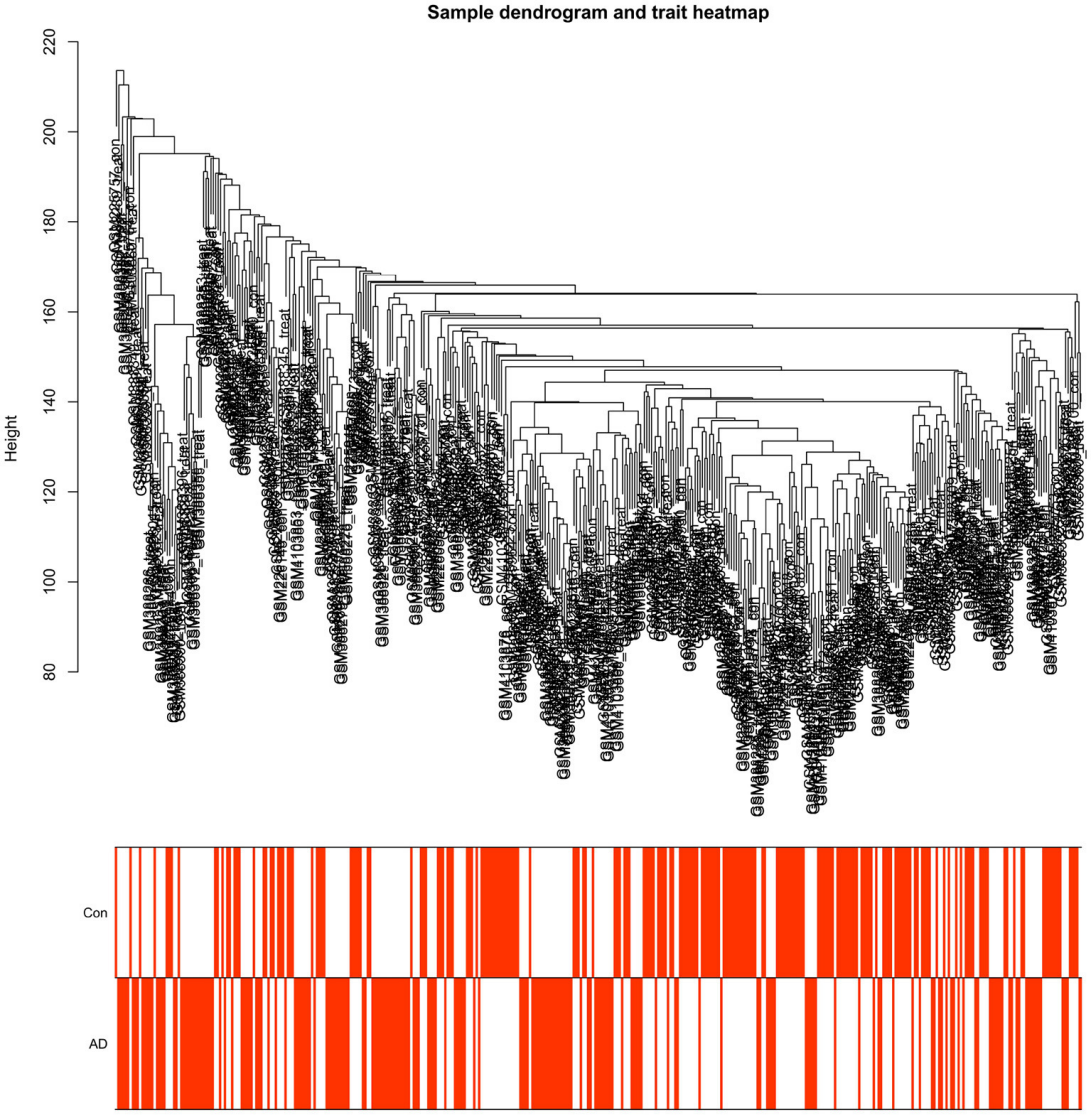
Clustering dendrogram of 399 samples.

We combined datasets GSE15222 and GSE138260 as a training group to build a model, and then used dataset GSE122063 as a test group to evaluate model accurateness. In the training group, the genes common to the two datasets were screened out, and the expression levels of each sample were combined into a matrix. The combined dataset had a total of 399 samples to be included in the model for subsequent analysis. Of them, 193 were AD-related and 206 were normal samples. For its part, the validation group had 66 AD samples and 44 normal samples in its gene expression matrix. The merged dataset was normalized using the “limma” package, and then we clustered the samples to avoid significant outliers. All samples in the combined dataset were included in our study (Figure 2).

### 3.2. Differential gene expression in the AD merged data

**Figure 3:**
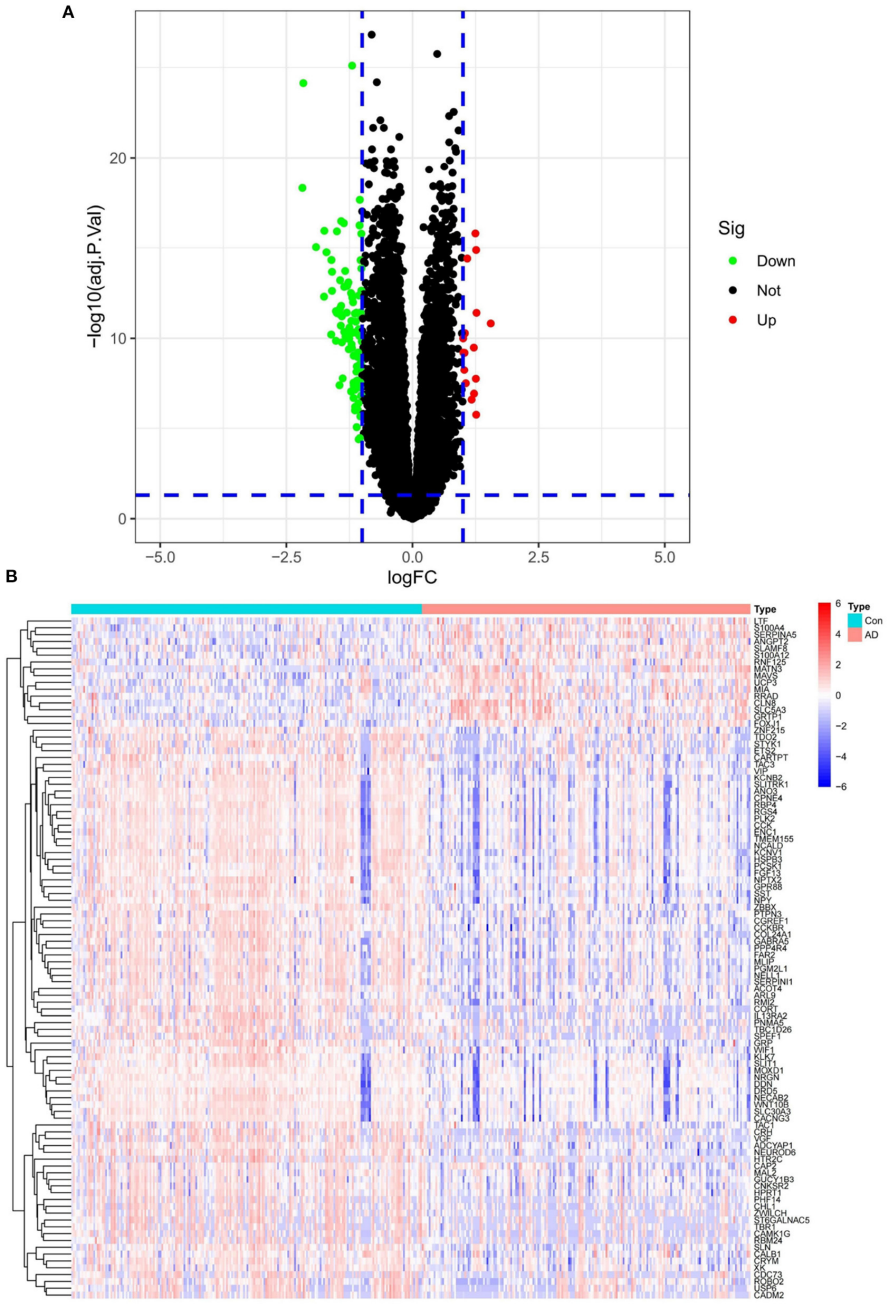
**(A)** Merged dataset differential gene volcano plot. **(B)** Merged dataset differential gene heatmap.

In the AD merged data, a total of 111 differential genes were detected, of which only 16 were up-regulated. The global results are presented in a volcano plot (Figure 3A). The expression changes are also shown in the form of a heatmap (Figure 3B).

### 3.3. GO and KEGG analysis of differential genes

**Figure 4:**
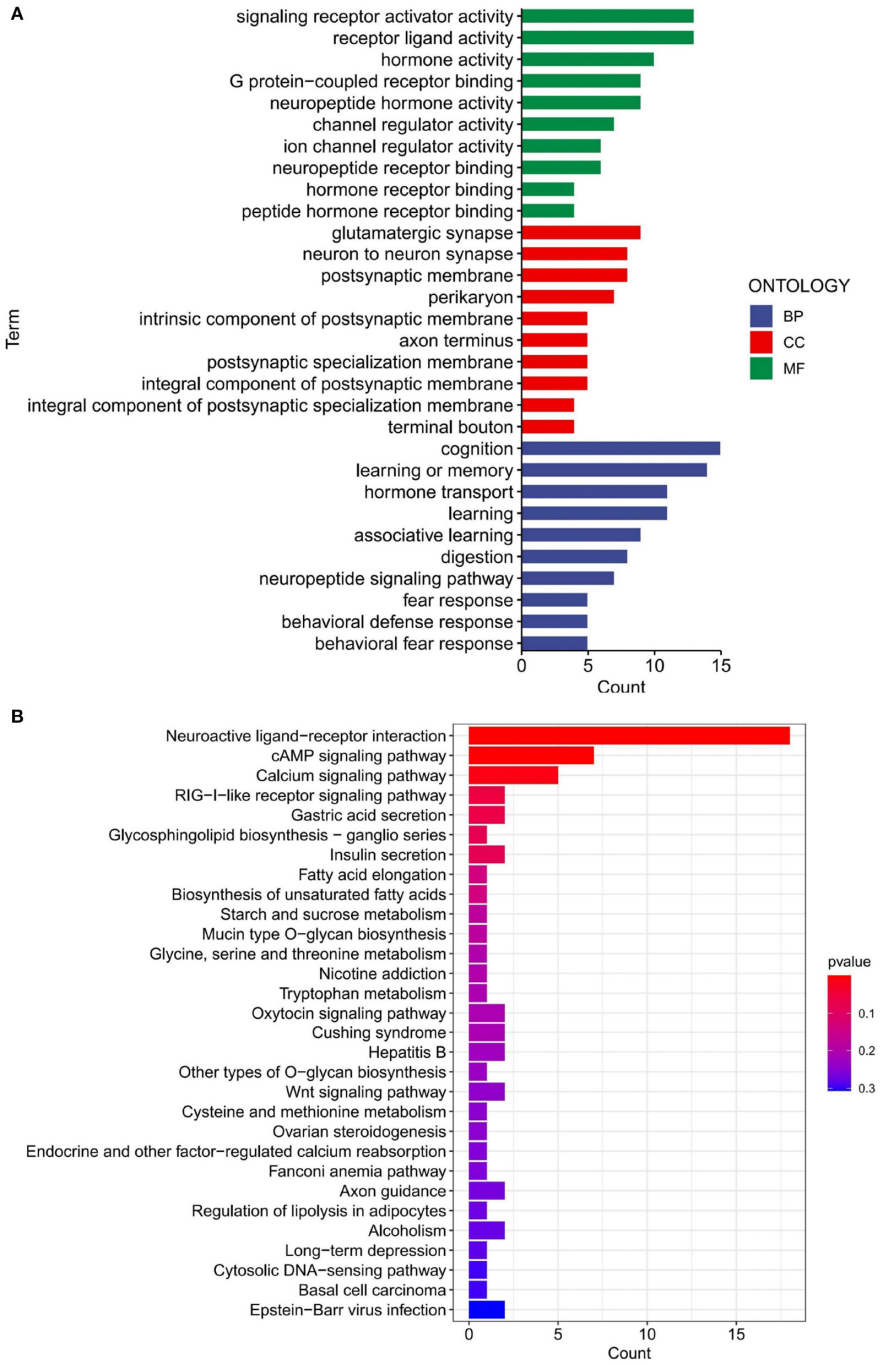
GO and KEGG analyses of merged dataset of differential genes. **(A)** GO analysis of merged dataset of differential genes. BP, biological process; CC, cellular components; MF, molecular function. **(B)** KEGG analysis of merged dataset of differential genes.

The results of the GO and KEGG enrichment analysis performed on the previously obtained 111 differential genes are depicted in Figure 4A. As shown for GO, BPs were mainly associated with response to cognition and learning or memory. The pathways concentrated in MF were signaling receptor activator activity and receptor-ligand activity. For CC, the most notable results were glutamatergic synapse, neuron to-neuron synapse, and postsynaptic membrane. On the other hand, the differential genes were enriched in three pathways: Neuroactive ligand-receptor interaction, cAMP signaling pathway, and Calcium signaling pathway, as displayed in the KEGG bar graph (Figure 4B). These enriched pathways are all related to cognition, learning, and memory, and in line with our overall picture that AD patients may lose cognitive and memory-related abilities. The present results suggest the relevance of these genes for our follow-up research.

### 3.4. Construction and identification of core modules through WGCNA

**Figure 5:**
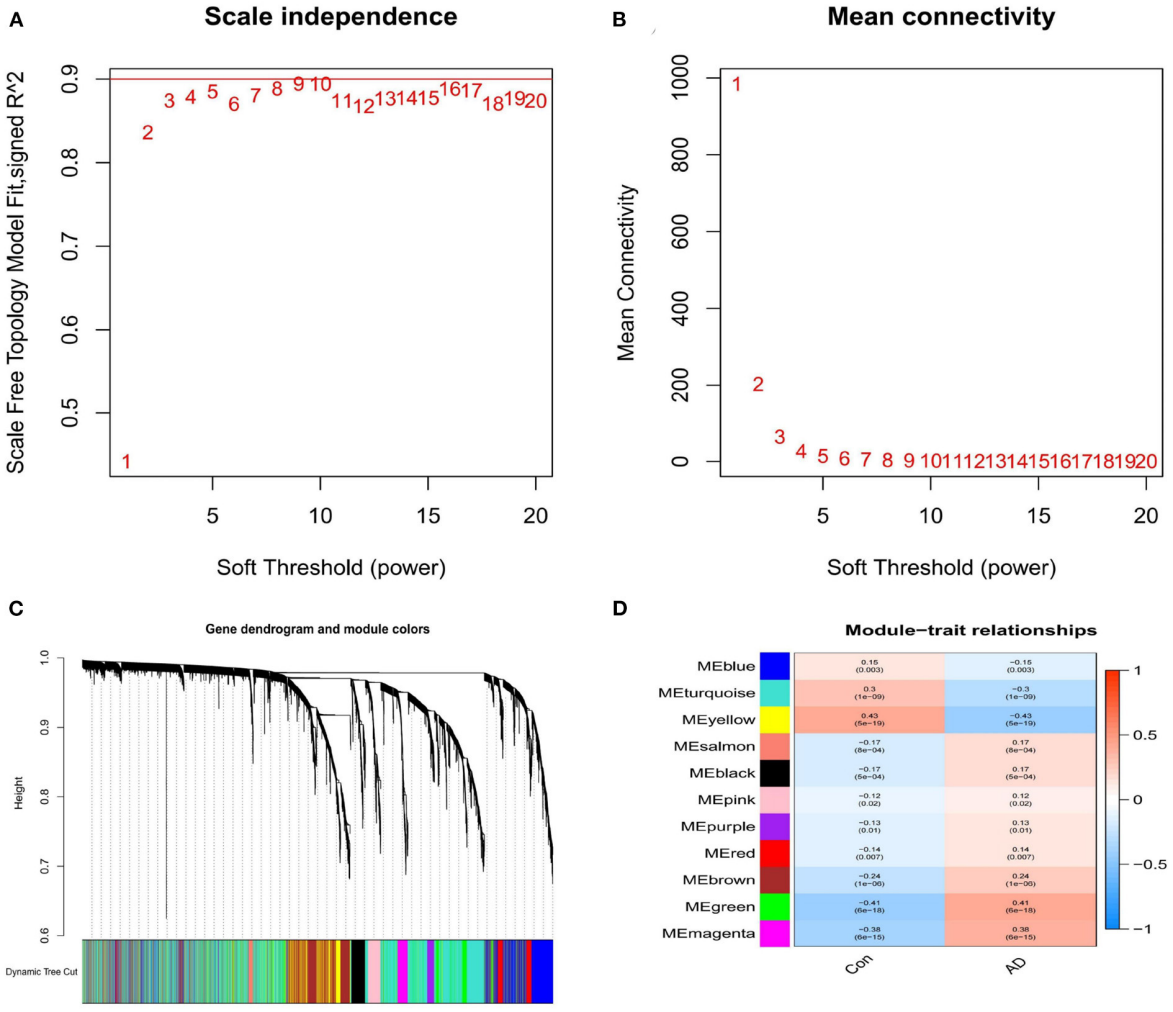
**(A)** Analysis of the scale-free index for various soft-threshold powers (β). **(B)** Analysis of the mean connectivity for various soft-threshold powers. **(C)** Identification of co-expression gene modules. **(D)** A heatmap showing the correlation between each module eigengene and phenotype.

A scale-free network was established with the WGCNA method, while a soft threshold was set to 9 by calculation (*R*^2^ = 0.86) (Figures 5A,B). The corresponding adjacency and topological overlap matrix were established, and all the genes in the merged dataset were clustered. A total of 11 modules were identified (Figure 5C, different colors). After a comprehensive analysis of the 11 modules, three of them showed to be highly correlated with AD. In particular, AD correlation values to each module were: 0.43 to the yellow module (*P* = 5e-19), −0.41 to the green module (*P* = 6e-18), and 0.38 to the magenta module (*P* = 6e- 15, Figure 5D). Fifty-one genes in the yellow, green and magenta modules were retained for further analysis.

### 3.5. Screening AD-related feature genes based on the LASSO algorithm

**Figure 6:**
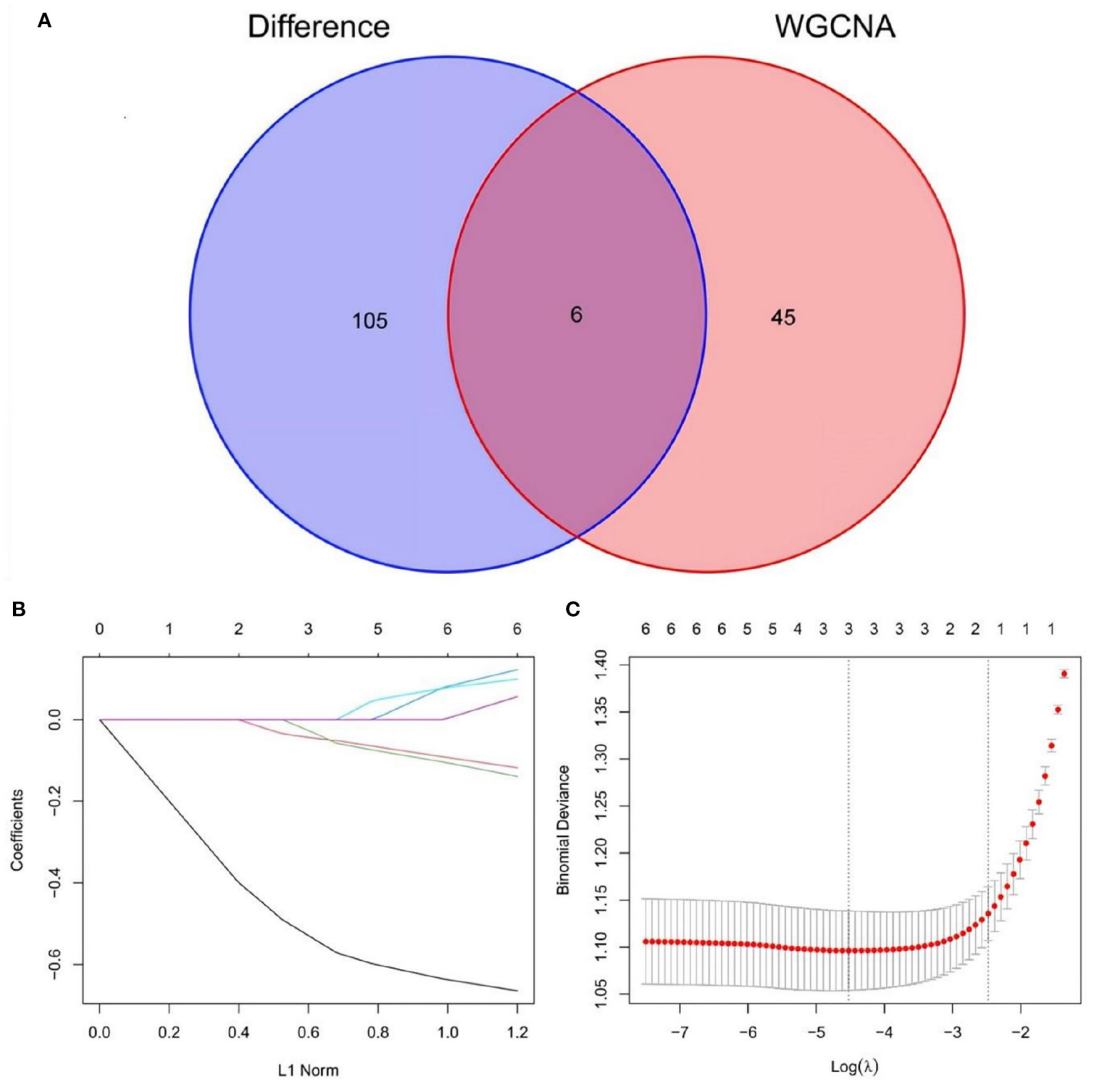
**(A)** Venn diagram show the intersection of differential genes from the merged dataset and hub genes derived from WGCNA. **(B,C)** LASSO regression screened the best AD-related feature genes.

**Figure 7:**
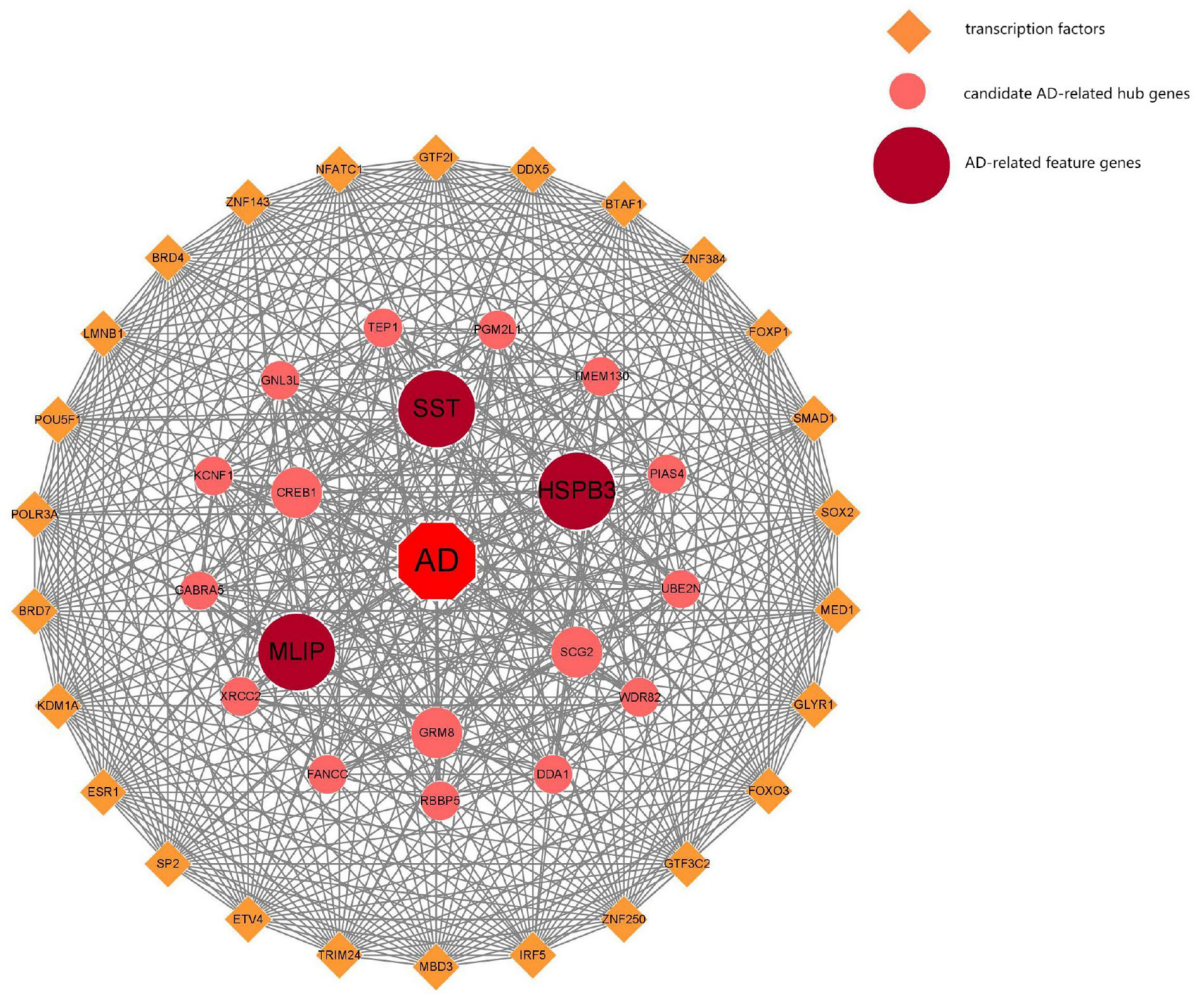
AD-related feature transcription factor analysis.

A total of 6 AD key genes were found by intersecting the 111 differential genes with the 51 module core genes obtained in the above WGCNA analysis (Figure 6A) SST, MLIP, HSPB3, PGM2L1, GABRA5, NCALD. Then, these six genes were filtered by the LASSO regression using the L1 criterion to obtain the feature genes of the disease. The resulting regression coefficient changes are shown in Figure 6B. A diagram showing the selection process of the cross-validation parameter λ was drawn. There, log(λ) was on the horizontal axis, while the root mean square error value was on the vertical axis (Figure 6C). When the value of the model variable was 3, the root means square error value was the smallest. This way, 3 AD-related feature genes are obtained: SST, MLIP, and HSPB3. The parameter values of these biomarker genes are shown in Table 1. And, transcription factor analysis is shown in Figure 7.

**Table 1:** The characteristics of the AD-related feature genes.

**Gene_symbol**	**Gene_name**	**RNA-seq**		

		**Fold change**	***P*** **-value**	**FDR**
SST	Somatostatin	−2.164	2.34E-28	7.32E-25
MLIP	Muscular LMNA-interacting protein	−1.746	9.61E-19	1.15E-16
HSPB3	Heat shock protein beta-3	−1.499	1.04E-18	1.22E-16

### 3.6. Identification and validation of AD-related feature genes

**Figure 8:**
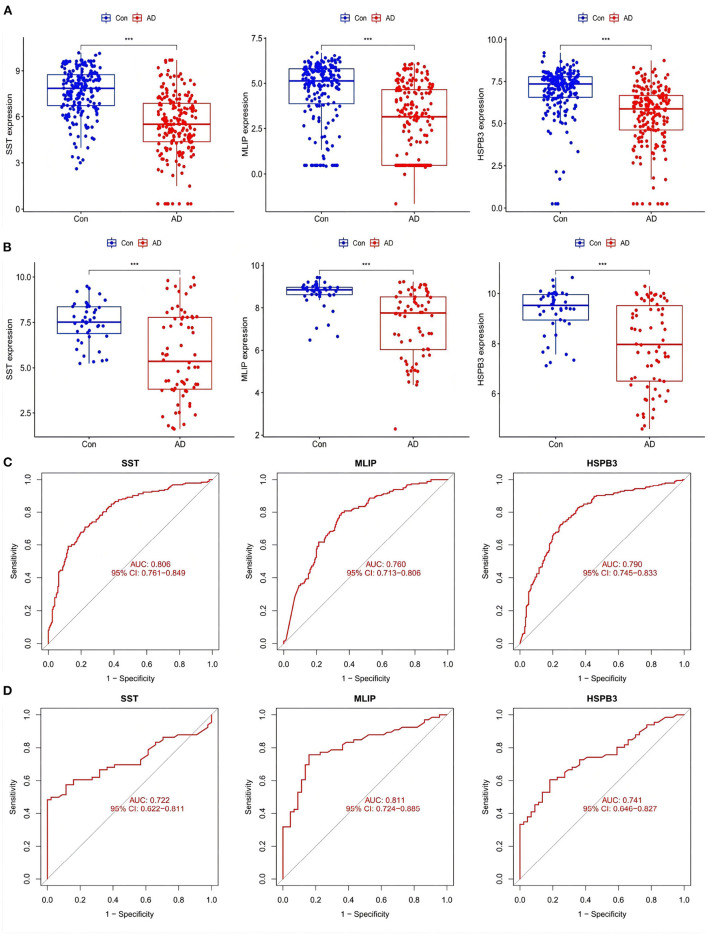
**(A)** Expression of AD-related feature genes in Training group. **(B)** Expression of AD-related feature genes in Training group. ***P <0.001. **(C,D)** ROC curves of AD-related feature genes in Training group and Test group.

After screening out AD-related feature genes by the LASSO regression, the difference boxplot of the Training group was constructed. This boxplot showed that the AD-related feature genes SST, MLIP, and HSPB3 had significant differences between the AD group and the normal group (*P* < 0.001, Figure 8A). These three disease feature genes were all down-regulated in the AD group samples.

Then, the boxplot of the Test group was used to validate SST, MLIP, and HSPB3 as AD-related feature genes. There were significant differences between the genes in the test group compared with the normal group (*P* < 0.001). In line with the Train group, the evaluated genes were all down-regulated (Figure 8B). The ROC curve displayed the accuracy in the diagnostic character of the screened genes. The corresponding area under the ROC curve (AUC) in both the Training and Test groups were greater than 0.7 (Figures 8C,D, respectively). The results suggested that SST, MLIP, and HSPB3 had high accuracy as AD diagnostic genes.

### 3.7. Correlation analysis between AD-related feature genes and immune cells

**Figure 9:**
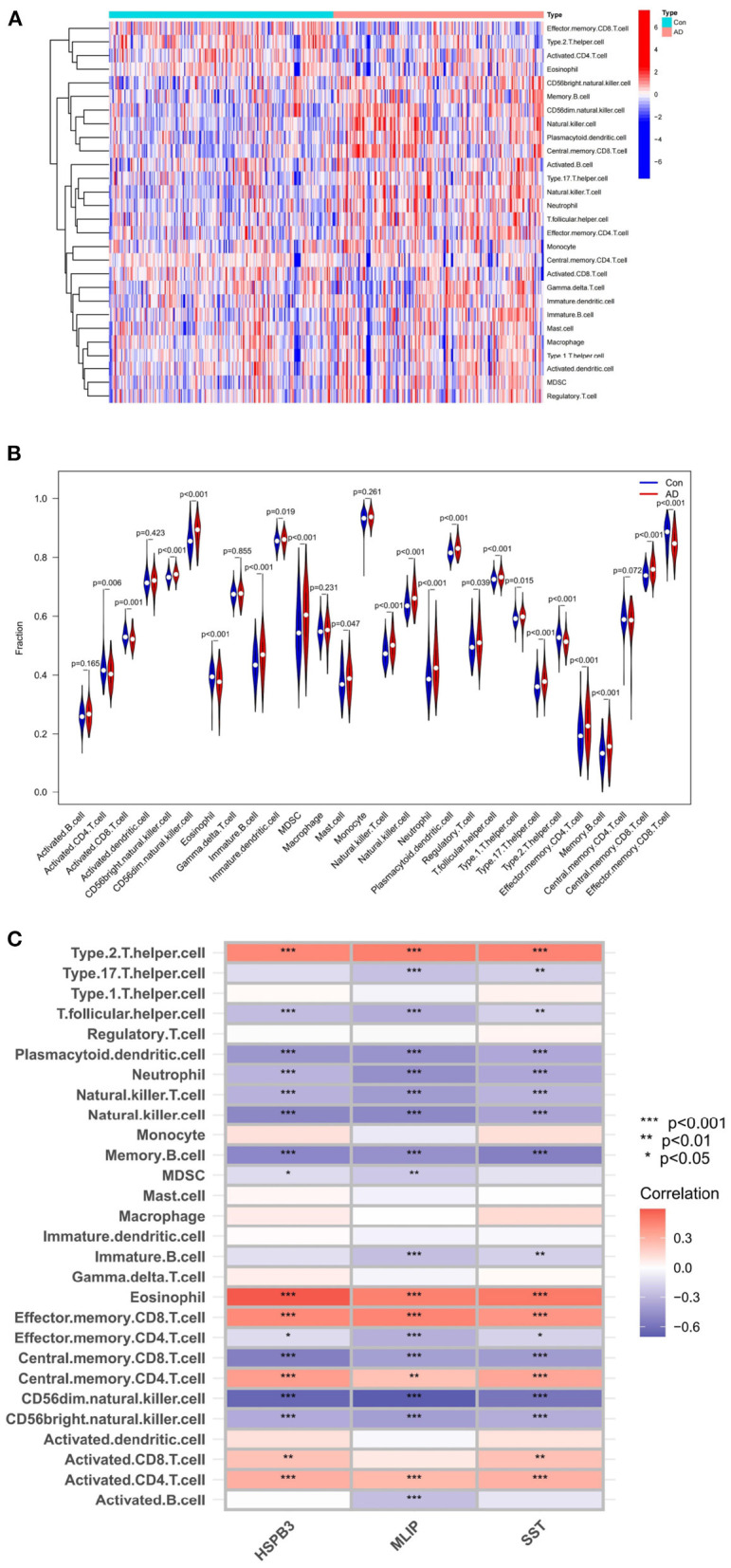
**(A)** Immune-related function heatmap. **(B)** violin diagram of AD-related differential immune cell. **(C)** Heatmap of correlations between AD-related feature genes and immune cells.

The ssGSEA analysis enabled the construction of an immune cell heatmap using the scores of each immune cell (Figure 9A). The abscissa represented the sample type (AD or Con), and the ordinate, the type of immune cells. Moreover, a violin plot of immune cell differences (Figure 9B) indicated a total of 16 immune cell differences between the normal and AD groups (*P* < 0.001). The abscissa of this violin plot denoted the name of each immune cell, and the ordinate, the content of each immune cell. The blue in the figure represented the samples of the normal group, while the red represented those of the AD group (Figure 9B). The results suggested that, in AD patients, the content of the immune cells was significantly different from that of the normal group. In these differential immune cells, Eosinophil, Type 2 T helper cell, and Effector memory CD8 T cell were down-regulated in AD patients. The rest of the immune cells were all up-regulated.

Based on the above analysis, a correlation analysis was conducted to evaluate which immune cells were correlated with AD-related feature genes. Subsequently, a corresponding heatmap was obtained (Figure 9C).

## 4. Conclusion

In summary, we identified three AD-related feature genes (SST, MLIP, HSPB3), which passed the verification in the Test group. These three genes may serve as AD markers and effective indicators for AD treatment.

## 5. Discussion

Currently, immune analysis for neurodegenerative diseases is gradually entering the big academic picture. The common notion is that immune cells cannot quickly access a healthy brain, which is true most of the time. However, recent research in humans and other species has found that immune cells not only can access a still-healthy aging brain, but can also reach the precise cerebral areas where new neurons are generated. This unlocks new directions for neurodegeneration research. Various neurological diseases have a close interaction with the immune system. In the human meninges, the boundary of the central nervous system, both innate and acquired immune cells are abundant. A different cytoimmunological situation is observed in the brain parenchyma. For the immune response to function properly, the brain parenchyma often adopts another strategy: considerable lymphocyte infiltration.

Interestingly, multiple studies have demonstrated that interventions on the immune system in the meninges can modulate the nervous system function and behavior. This regulation depends on neuron-specific receptor signaling, for instance, on T cells and their secreted cytokines (Derecki et al., 2010; Filiano et al., 2016; Lima et al., 2020). All the above implicates that the immune activity in the meninges is crucial for the brain's normal functioning. As a result, the classic idea that the brain is an immune-privileged organ is challenged.

At the present, the common academic idea is that cerebrospinal fluid antigens first reach the neck lymph nodes and then enter the lymphatic system. This process requires an immune system network in the brain, which consists of the lymph in the meninges and the cerebrospinal fluid (Mrdjen et al., 2018; Hove et al., 2019), as has been demonstrated in autoimmune encephalomyelitis, glioblastoma and other diseases. However, the specific anatomical sites and cellular mechanisms of antigen presentation and activation of T cells in the central nervous system remain poorly understood.

We have previously identified the dural sinuses as the site of neuroimmune interactions in the brain, where antigens in the cerebrospinal fluid are recognized by antigen-presenting cells and signal to the locally-enriched T cells. Moreover, endothelial and parietal cells in the dural sinuses would also play an auxiliary role in this process. Therefore, the dural sinus network would have a crucial role in the normal functioning of the brain as well as in pathological conditions, and the intervention of this cerebral location may become part of new treatments to alleviate neurological diseases (Rustenhoven et al., 2021).

In this study, three AD-related feature genes (SST, MLIP, and HSPB3) were identified by WGCNA analysis and subsequent LASSO regression. Somatostatin (SST) is released in the hypothalamus and, as the name suggests, inhibits growth by preventing the release of growth hormones from the pituitary gland. A previous study on the SST gene revealed that the content of somatostatin in the cerebrospinal fluid of patients with vascular dementia was significantly lower than in a healthy person. We compared the data of patients with cognitive impairment and found that both diseases are accompanied by a decline in this gene, which would be one of the causes of dementia in patients with cerebral infarction.

In the present study, the content of SST was also decreased in the AD group. Thus, the down-regulation of this gene may contribute to the occurrence and development of AD. Nevertheless, more evidence is needed to support this hypothesis. In addition, related studies have proposed that SST is an important neuromodulator in the dentate gyrus, and disruption of the associated signaling system may significantly impact hippocampal function. The latter may have established a more vital link between this gene and AD.

On the other hand, the MLIP gene is often reported to be associated with muscle, and enriched in class A lamina-interacting protein. This is a unique protein required for normal cardiac muscle adaptation to stress (Cattin et al., 2015). Recently, the MLIP gene was identified as responsible for rhabdomyolysis, and decreased overall RNA expression levels of major MLIP isoforms were observed in the skeletal muscle of patients. MLIP is now identified as a novel disease-associated gene in humans, with a role in normal and diseased skeletal muscle homeostasis (Osorio et al., 2021). In particular, the influence of the MLIP gene in AD development is yet to be elucidated.

Finally, HSPB3 is one of the small human heat shock proteins, with a molecular chaperone family of 10 members (HSPB1-HSPB10). HSPB mutations may have serious consequences, such as peripheral neuropathy caused by point mutations in HSPB1 and HSPB8. Thus, HSPB3 research requires special attention. In addition, a new missense mutation in HSPB3 (R7S) has been recently identified in axonal motor neuropathy. A further article that employed bioinformatics methods also identified HSPB3 as a central gene associated with AD (Jiang et al., 2021). Lastly, this gene has also been reported to cause motor neuropathy.

## Data availability statement

The original contributions presented in the study are included in the article/supplementary material, further inquiries can be directed to the corresponding author/s.

## Author contributions

JZ designed the study, oversaw the interpretation of the results, and revised the manuscript. HS and PH performed data analysis and drafted and revised the manuscript. JY and XL conducted the literature review. YL, ZX, HH, XT, TJ, SD, and CZ completed the remaining tasks of writing the article. All authors read and approved the final manuscript.

## Funding

This study was supported by the Fund for Shanxi 1331 Project (2021-5-2-2-B1); the Natural Science Foundation for Youths of Shanxi Province, China (20210302124301); the Provincial Science and Technology Grant of Shanxi Province (20210302124588); Changzhi Medical College Innovation Team (CX202001).

## Conflict of interest

The authors declare that the research was conducted in the absence of any commercial or financial relationships that could be construed as a potential conflict of interest.

## Publisher's note

All claims expressed in this article are solely those of the authors and do not necessarily represent those of their affiliated organizations, or those of the publisher, the editors and the reviewers. Any product that may be evaluated in this article, or claim that may be made by its manufacturer, is not guaranteed or endorsed by the publisher.
